# Liver steatosis in Chinese HIV-infected patients with hypertriglyceridemia: characteristics and independent risk factors

**DOI:** 10.1186/1743-422X-10-261

**Published:** 2013-08-14

**Authors:** Jiang Xiao, Ning Han, Di Yang, Hongxin Zhao

**Affiliations:** 1 The Center of Infectious Diseases, Ditan Hospital, Capital Medical University, Beijing 100015, China

**Keywords:** HIV, HAART, Liver steatosis, Hypertriglyceridemia

## Abstract

**Background:**

Since Highly Active Antiretroviral Therapy (HAART) medications were made available in 2002, multiple serious side effects have been observed. However, no study has yet systematically evaluated the prevalence of liver steatosis, a very serious but treatable side effect.

**Objectives:**

This study examined the prevalence of and independent risk factors for liver steatosis in Chinese HIV-infected, HAART-experienced patients who had been diagnosed with hypertriglyceridemia.

**Methods:**

In this cross-sectional observational study, the prevalence of liver steatosis was determined by ultrasound imaging that detected diffusion in hepatic echogenicity. The risk factors associated with steatosis were evaluated with a proportional odds logistic regression model.

**Results:**

Among 163 HIV-infected patients with hypertriglyceridemia and past HAART experience, 75(46%) patients were determined to have liver steatosis. In multivariable logistic regression model, the risk factors associated with liver steatosis were: higher triglyceride level (OR = 1.086, *P* = 0.026), metabolic syndromes (OR = 2.092, *P* = 0.024) and exposure to nucleoside reverse transcriptase inhibitor (NRTIs) ((OR = 2.11, *P* = 0.001) and Stavudine (OR = 3.75, *P* = 0.01)). Exposure to Nevirapine (OR = 0 .41, *P* = 0.003) was a favorable factor for lipid metabolism *in vivo* and was a protective factors for liver steatosis.

**Conclusions:**

Chinese HIV-infected patients with hypertriglyceridemia appear to be prone to liver steatosis, especially those on NRTIs. Routine screening should be considered on their lipid panels.

## Background

Liver steatosis has become the most common non-viral hepatic disease worldwide [1]. In developed countries, prevalence of nonalcoholic fatty liver disease(NAFLD) fluctuates between 20-30% in the general population [1,2]; in the U.S., it is between 17-33% [3-5]. Fan Jiangao et al. [6] reported that the prevalence of liver steatosis was 15% in Shanghai general population and gradually increasing. Studies have shown that risk factors for liver steatosis include obesity, dyslipidemia, diabetes, insulin resistance and other factors.

Among the HIV-infected population, the widespread use of HAART has reduced mortality and increased survival rates of AIDS patients but has also brought with its metabolic side effects, such as hyperlipidemia and fatty liver (i.e. steatosis). Crum-Cianflone et al. [7] reported that prevalence of NAFLD in HIV-infected population in the U.S. Veterans Administration Health System was 31% based on ultrasound imaging, with a greater waist circumference, lowered high-density lipoprotein (HDL) and elevated triglyceride levels. Lesi et al. [8] reported that 13.3% of Africans with HIV had elevated low-density lipoprotein (LDL) levels.

In China, the pilot for the National Free HAART Program began in Henan Province in 2002, and the program fully began in 2003 [9]. The extent of liver steatosis has yet to be determined. In the present study, we examined the prevalence of and risk factors associated with liver steatosis among a cohort of Chinese HIV-infected patients with hypertriglyceridemia.

## Methods

The study was received the approval of the Ethics Committee from following study sites: Ditan and You’an Hospital in north China, Henan Traditional Chinese medicine (TCM) Hospital and Anhui TCM Hospital in central China, Guangzhou 8th Hospital in South China and Xiangya Second Hospital in south-central China. All of the participants meeting the inclusion criteria had signed informed consent.

### Procedures

We conducted a cross-sectional observational study. According to *guideline for prevention and treatment of AIDS in China*[10] and *guideline for prevention and treatment of hyperlipidemia in adults*[11] recommended by Chinese Medical Association, eligible participants in HIV-infected patients with hypertriglyceridemia were: (1) HIV-positive (confirmed by enzyme-linked immunosorbent assay (ELISA) and Western Blot), (2) HAART experienced, (3) and diagnosed with hypertriglyceridemia(marginal increase (1.70-2.25 mmol/L) or an increase (>2.26 mmol/L)) [11]. Patients with severe primary disease of the heart, liver, kidney or hematopoietic systems, taking lipid-lowering drugs, or who were pregnant or breast-feeding were excluded. Patients were recruited and enrolled at Ditan and You’an Hospitals in north China, Henan and Anhui Traditional Chinese Medicine (TCM) Hospitals in central China, Guangzhou 8^th^ Hospital in south China, and Xiangya Second Hospital in south-central China. This study was approved by Institutional Review Boards at each hospital, and all enrolled participants provided written informed consent.

The study was conducted from January 2008 to June 2010 as part of a parent study on side effects treatment. Across study sites, 180 participants were recruited and scheduled for ultrasound imaging. As 17 did not appear for the screening visit, data were available for 163 participants.

HIV/HCV co-infected patients and normal control participants have also been enrolled as control groups in this study. eligible participants in HIV/HCV co-infected patients were: (1) HIV-positive, (2) HAART experienced, (3) positive serum antibodies to HCV and detectable serum HCV RNA. Exclusion criteria were: co-infection with HBV, decompensated cirrhosis and other known causes of liver disease. A total of 69 HIV/HCV co-infected patients met these criteria. Another 30 participants were enrolled as normal control.

### Measures

Participants completed a face-to-face paper-and-pencil questionnaire eliciting data on gender, age, ethnicity, smoking and drinking history (alcohol intake of more than 20 g/day for men or 10 g/day for women), time since HIV diagnosis, source of HIV transmission, time on HAART, and current CD4 count. Study staffs recorded participants’ height, weight, heart rate, blood pressure, and body mass index (BMI) as obese (≥ 30 kg/m^2^), overweight (25.0-29.9 kg/m^2^) and normal (18.6-24.9 kg/m^2^). Laboratory tests were performed for lipid panels as well as liver and kidney function (see Table 1); fasting plasma glucose; and current CD4 count. Metabolic syndrome was determined according to diagnostic criteria recommended by the Chinese Medical Association [11], i.e., meeting three or more of the following: BMI ≥ 25 kg/m^2^, serum triglyceride (TG) ≥ 1.7 mmol/L, serum lower HDL (men ≤ 0.91 mmol/L, women ≤1.04 mmol/L), blood pressure ≥140/90 mmHg, fasting plasma glucose ≥6.1 mmol/L, and diabetes.

**Table 1:** Baseline descriptive data for study centers in Chinese HIV patients with hypertriglyceridemia

**Baseline descriptive data**	**Cases (%)**
**Study center (total 180 cases)**	
Ditan Hospital (north China)	42 (23.3%)
You’an Hospital (north China)	24 (13.3%)
Henan TCM Hospital (central China)	48 (26.7%)
Anhui TCM Hospital (central China)	18 (10.0%)
Guangzhou 8^th^ Hospital (south China)	30 (16.7%)
Xiangya 2^nd^ Hospital (south-central China)	18 (10.0%)

All participants underwent an ultrasound to detect liver steatosis and hepatomegaly. Per Chinese Medical Association recommendations [12], liver steatosis was defined by ultrasound imaging indicating diffusion in hepatic echogenicity.

### Statistical analysis

All data were analyzed using SAS 9.1.3 (SAS Institute, Cary, NC, USA). Continuous variables were expressed by mean ± standard deviation and Analysis of Variance was used for comparing quantitative variables among or between normal control, HIV/HCV co-infection group and HIV-infected patients with hypertriglyceridemia, respectively. Categorical variables were expressed by percentages and chi-squared tests were used for statistical comparisons of categorical variables. *P* < 0.05 was considered significant.

In HIV-infected patients with hypertriglyceridemia, univariate logistic regression models were first used to determine the association of the following variables with the presence of liver steatosis: sex; age; drinking history; time since HIV diagnosis; time on HAART; blood lipid panels (total cholesterol, total triglycerides, HDL and LDL levels); metabolic syndrome, liver and renal functions (ALT, AST, T-BIL, D-BIL, BUN, creatinine); fasting plasma glucose; and current CD4 cell counts. Body mass index (BMI) is graded and has been shown for female vs male, a chi-square test was firstly used to determine if BMI was associated with the presence of liver steatosis. Statistically significant predictors were included in a subsequent multivariate logistic regression model.

In HIV-infected patients with hypertriglyceridemia, a chi-square test was used to determine if antiretroviral medication was associated with the presence of liver steatosis and then all medications were entered into a multivariate logistic regression analysis to determine their unique association with liver steatosis. Alpha was set to 0.05, with 95% confidence intervals.

## Results

### Participant characteristics and baseline descriptive data in HIV-infected patients with hypertriglyceridemia

In this study, 180 HIV-infected patients with hypertriglyceridemia and past HAART experience have been recruited in several AIDS Treatment Centers in China (Table 1). As 17 did not appear for the screening visit, data were available for 163 participants.

See Tables 1 and 2 for a full description of 163 HIV-infected patients with hypertriglyceridemia. As seen in Table 2, mean age was 42.9 ± 9.3 years; 109 (66.9%) were male. average time since HIV diagnosis and time on HAART were 132 months (SD = 73 months) and 38.7 months (SD = 25.3 months), respectively. Mean CD4 count was 316 cells/ul.

**Table 2:** Characteristics and independent risk factors of liver steatosis in Chinese HIV patients with hypertriglyceridemia analyzed with univariate and multivariate regression analysis

				**Univariate analysis**		**Mutivariate analysis**	
**Charateristics**	**All patients**	**Liver steatosis**	**Non-steatosis**	**OR**	***P *value**	**OR**	***P *value**
**Features**							
Age (yr)	42.9 ± 9.3	43.2 ± 9.5	42.9 ± 9.2	0.99	0.79		
Male	109 (66.9%)	52 (31.9%)	57 (35.0%)	0.47	**0.02**		
Female	54 (33.1%)	36 (22.1%)	18 (11.0%)				
Smoking histroy	52 (31.9%)	27 (30.7%)	25 (33.3%)	1.13	0.72		
Drinking	17 (10.4%)	12 (13.6%)	5 (6.7%)	4.615	**0.03**		
**BMI (Kg/m**^**2**^**)**							
Male patients							
25-29.9	76 (69.7%)	41 (37.6%)	35 (32.1%)	0.68	0.71		
≥30	13 (11.9%)	6 (5.5%)	7 (6.4%)				
18.6-24.9	20 (18.4)	11 (10.1%)	9 (8.3%)				
Female patients							
25-29.9	36 (66.7%)	12 (22.2%)	24 (44.5%)	**<0.001**	1.00		
≥30	6 (11.1%)	2 (3.7%)	4 (7.4%)				
18.6-24.9	12 (22.2%)	4 (7.4%)	8 (14.8%)				
**Biochemistry tests**							
ALT (U/L)	32.5 ± 29.3	28.8 ± 25.7	36.7 ± 32.7	1.01	0.11		
AST (U/L)	31.7 ± 30.9	37.7 ± 32.1	31.3 ± 21.5	0.99	0.78		
CK	115 ± 112	131 ± 186	109 ± 62	0.99	0.44		
BUN (mmol/L)	4.9 ± 1.4	4.8 ± 1.0	4.9 ± 1.8	1.04	0.55		
Cr (umol/L)	77.0 ± 28.5	82.3 ± 18.7	70.9 ± 35.9	0.97	0.01		
**Blood lipid levels**							
TG (mmol/L)	5.7 ± 5.2	4.7 ± 3.8	6.9 ± 6.3	1.10	**0.012**	1.086	**0.026**
TC (mmol/L)	5.5 ± 1.7	5.3 ± 1.7	5.8 ± 1.7	1.19	0.07		
LDL (mmol/L)	2.8 ± 1.0	2.9 ± 0.9	2.7 ± 1.1	0.83	0.31		
HDL (mmol/L)	1.3 ± 0.3	1.3 ± 0.3	1.2 ± 0.4	0.37	0.08		
FPG (mmol/L)	5.7 ± 1.8	5.1 ± 1.2	6.5 ± 2.1	1.90	**<0.001**		
**Metabolic sydrome**	78 (47.9%)	34 (38.6%)	44 (58.7%)	2.25	**0.01**	2.092	**0.024**
**HIV infection**							
Time since HIV	132 ± 73	141 ± 74	117 ± 69.5	0.99	0.07		
diagnosis (M)							
Mos. on ART (M)	38.7 ± 25.3	37.5 ± 25.3	40.1 ± 25.5	1.00	0.39		
Current CD4	316 ± 191	240 ± 141	401 ± 204	1.01	**<0.001**		

**Notes:***BMI* body metabolic index, *ALT* Alanine aminotransferase, *AST* Aspartate aminotransferase, *CK* Creatine kinase, *BUN* Blood urea nitrogen, *Cr* Serum creatinine, *TG* triglyceride, *TC* Total cholesterol, *LDL* Low-density lipoprotein, *HDL* high-density lipoprotein, *FPG* Fasting plasma glucose.

The study population was 100% Han Chinese (the majority ethnic group). In 163 patients, 150 were taking HAART and 13 had temporary discontinued ARV regimens due to resistance. Participants had started on first-line recommended HAART regimens [9] AZT/d4T + 3TC + NVP/EFV; 17 (9.8%) had been on a second-line HAART regimen, TDF + 3TC + LPV/r.

There was a marginal increase (5.18-6.19 mmol/L) or an increase (>6.22 mmol/L) in total cholesterol levels in 39 (23.9%) and 45 (27.6%) participants, respectively; a marginal increase (3.37-4.12 mmol/L) or increase (>4.14 mmol/L) in LDL levels were detected in 24 (14.7%) and 14 (8.6%) patients, respectively; 34 (20.9%) presented with lowered HDL (<1.04 mmol/L). Biochemistry tests indicated that liver and renal functions were normal results except for a mild elevation of ALT (ALT > 40 U/L) found in 36 patients (22.6%).

### Metabolic changes in HIV-infected patients with hypertriglyceridemia: comparisons with HIV/HCV Co-infected patients and normal control participants

Metabolic changes in this study was illustrated in Table 3, showing that 3 groups of participants (HIV with hypertriglyceridemia, HIV/HCV co-infection and normal control) did not differ by age and percentage of genders. Cholesterol serum levels and prevalence of hypercholesterolemia were significantly higher in HIV-infected patients with hypertriglyceridemia than that HIV/HCV co-infected patients and normal control participants (p < 0.0001). We also found that HDL serum levels were 1.2 ± 0.3 and 0.9 ± 0.4 mmol/L in patients with hypertriglyceridemia and HIV/HCV co-infection, which were significantly lower than that in normal control (*p* < 0.0001), and prevalence of lower HDL serum levels was similar in patients with hypertriglyceridemia and control participants (20.9% & 23.3%) but was significantly higher in HIV/HCV co-infected patients.

**Table 3:** Metabolic changes in HIV-infected patients with hypertriglyceridemia: comparsion with HIV/HCV co-infected patients and normal control participants

**Characteristics**	**Control**	**HIV/HCV co-infection**	**HIV-infected patients with hypertriglyceridemia**	***p *value**
Age (yr)	42.0 ± 4.3	42.7 ± 6.1	42.9 ± 9.3	0.873
Male (%)	14 (46.7%)	47 (68.1%)	109 (66.9%)	0.104
Female (%)	16 (53.3%)	22 (31.9%)	54 (33.1%)	
**Transmission route**				
Former plasma donators	--	14 (20.3%)	41 (25.2%)	--
Blood transfusion	--	39 (59.5%)	9 (5.5%)	--
Intravenous drug users	--	7 (7.2%)	20 (12.3%)	--
Sexual transmission	--	9 (13.0%)	89 (54.6%)	--
Unknown reason	--	0 (0.0%)	4 (2.5%)	--
**Biochemistry tests**				
ALT (U/L)	18.7 ± 10.9	44.3 ± 40.3	32.5 ± 29.3	**0.001**
T-BIL (umol/L)	11.1 ± 3.9	12.7 ± 9.2	11.4 ± 6.2	0.129
BUN (mmol/L)	4.7 ± 1.1	4.8 ± 3.9	4.9 ± 1.4	0.836
FPG (mmol/L)	5.5 ± 1.0	5.7 ± 1.7	5.7 ± 1.8	0.755
**Blood lipid levels**				
TG (mmol/L)	1.9 ± 1.7	1.8 ± 1.1	5.7 ± 5.2	**<0.001**
TC (mmol/L)	4.9 ± 0.9	3.7 ± 1.1	5.5 ± 1.7	**<0.001**
LDL (mmol/L)	2.8 ± 0.7	2.2 ± 0.9	2.8 ± 1.0	**<0.001**
HDL (mmol/L)	1.4 ± 0.7	0.9 ± 0.4	1.2 ± 0.3	**<0.001**
TG ≥2.26 mmol/L (%)	4 (13.3%)	12 (17.4%)	163 (100%)	**<0.001**
1.70 ≤ TG ≤ 2.25 mmol/L	6 (20.0%)	11 (15.9%)	0 (0%)	
TC ≥6.22 mmol/L	2 (6.7%)	2 (2.9%)	45 (27.6%)	**<0.001**
5.18 ≤ TC ≤ 6.19 mmol/L	10 (33.3%)	2 (2.9%)	39 (23.9%)	
LDL ≥4.14 mmol/L	0 (0.0%)	2 (2.9%)	14 (8.6%)	**0.041**
3.37 ≤ LDL ≤ 4.14 mmol/L	7 (23.3%)	5 (7.2%)	24 (14.7%)	
HDL ≤ 1.04 mmol/L	7 (23.3%)	50 (72.4%)	34 (20.9%)	**<0.001**
**Metabolic sydrome**	5 (16.7%)	13 (18.9%)	78 (47.9%)	**<0.001**
**Liver steatosis**	5 (16.7%)	14 (20.3%)	75 (46%)	**<0.001**

**Note**: Across study sites, 180 HIV-infected Patients with Hypertriglyceridemia were recruited and scheduled for ultrasound imaging. As 17 did not appear for the screening visit, data were available for 163 participants.

Prevalence of metabolic syndrome was 47.9% in patients with hypertriglyceridemia while it was 18.9% and 16.7% in HIV/HCV co-infected patients and normal control, respectively. We also found that, in HIV-infected patients with hypertriglyceridemia, Prevalence of liver steatosis was significantly higher than that in HIV/HCV co-infected patients and normal control (p < 0.0001).

### Prevalence and predictors of liver steatosis in HIV-infected patients with hypertriglyceridemia

Liver steatosis was confirmed in 75 (46%) of the participants and 9 (5.5%) had hepatomegaly in this group. As seen in Table 2, univariate models indicated that gender, drinking history, triglyceride levels, high fasting plasma glucose, metabolic syndrome, and CD4 count was associated with liver steatosis. None of the other predictor was significantly associated with liver steatosis. In the multivariate analysis with these predictors, only total triglyceride levels (OR = 1.086, *P* = 0.026) and metabolic syndrome (OR = 2.092, *P* = 0.024) remained associated with liver steatosis.

Overall, HAART medications were significantly associated with the presence of liver steatosis, (χ^2^ = 11.07, *P* = 0.011). Further multivariate logistic regression analysis indicated that AZT (OR = 2.11, *P* = 0.001) and d4T (OR = 3.75, *P* = 0.01) were highly associated with greater likelihood of liver steatosis; while NVP (OR = 0.41, *P* = 0.003) was a protective factor (Table 4).

**Table 4:** Independent risk factors in ART regimens for liver steatosis in Chinese AIDS patients with hypertriglyceridemia analyzed with multivariate logistic regression analysis

						**Mutivariate analysis**	
**HIV medications**	**All patients**	**Non-steatosis**	**Steatosis**	**χ**^**2**^	***P *value**	**OR**	***P *value**
AZT	73 (44.8%)	32 (36.4%)	41 (54.7%)	11.07	**0.011**	2.11	**0.001**
d4T	66 (40.5%)	39 (44.3%)	27 (36.0%)			3.75	**0.01**
EFV	55 (33.7%)	19 (21.6%)	36 (48.0%)			3.35	0.07
NVP	78 (47.9%)	52 (58.0%)	37 (36.0%)			0.41	**0.0003**
Others	17 (9.8%)	9 (10.2%)	8 (9.3%)			0.90	0.36

**Notes**: *AZT* Zidovudine, *d4T* Stavudine, *EFV* Efavirenz, *NVP* Nevirapine.

## Discussion

The results of the present study, conducted in a cohort of Chinese HIV patients with hypertriglyceridemia, HIV/HCV co-infection and normal control, showed that liver steatosis was significantly more common in patients with hypertriglyceridemia than that in HIV/HCV co-infection and normal control. We found that the prevalence of liver steatosis was 16.7% in normal control participants, which was consistent with that reported in the general population by Fan et al. [6]. HIV/HCV co-infection may be at risk of developing steatosis and prevalence of liver steatosis in HIV/HCV co-infection in American and French were 69% [13] and 67% [14], respectively, while Qingnian et al. [15] reported that prevalence in Chinese HIV/HCV co-infected patients was 18.3%. In this study, we found the prevalence of liver steatosis was 20.3% in the cohort of HIV/HCV co-infection, which indicated that prevalence in Euro-American was much higher than that in Chinese HIV/HCV co-infected patients, and may be associated with genetic background and HCV subtype, such as HCV-3, in Euro-American.

This Chinese cohort study found that liver steatosis was a common condition of HIV patients with hypertriglyceridemia and was associated with male gender, metabolic syndrome, higher triglyceride levels, and higher fasting plasma glucose count as well as certain antiretroviral medications.

We found that high triglyceride levels were associated with liver steatosis in a Chinese HIV-infected population with hypertriglyceridemia. HIV-infected patients on HAART were at particular risk for liver steatosis. This may be due to a “two-hit” pathogenic mechanism: (1) HIV-infected patients had high rates of lipid and glucose abnormalities and triglyceride accumulation in liver; (2) Those with NRTIs in ARV regimens were further associated with lipid metabolic dysfunction, resulting in abnormal triglyceride accumulation in the liver. We found that some antiretroviral drugs were independent risk factors for liver steatosis in HIV-infected, HAART-experienced patients who had high baseline triglyceride levels.

We also found that metabolic syndrome was a further independent risk factor for liver steatosis in Chinese HIV-infected patients with hypertriglyceridemia. Liver steatosis appears to be a result of long-standing insulin-resistance and represents a serious hepatic manifestation of metabolic syndrome. After administering HAART, HIV-infected patients have been found to have lipid metabolic dysfunctions and lipid accumulation in the liver have resulted in insulin-resistance [16]. HIV-infected patients should be monitored for metabolic syndrome and liver steatosis after administering HAART, particularly if they have high triglyceride levels.

In this study, we found that NRTIs such as AZT and d4T were independent risk factors for liver steatosis, similar to results reported by Guaraldi et al. [16]. Few HIV patients in China are on protease inhibitors (PIs), but others have found both NRTIs and PIs to be associated with hyperlipidemia and liver steatosis [17,18]. NRTIs can inhibit mitochondrial polymerase γ and destroy lipid metabolic processes intracellularly, resulting in lipid accumulation *in vivo* and hyperlipidemia. HAART medications, especially thymidine analogs [19], have promoted insulin resistance [20] and are associated with liver steatosis *in vivo.*

We found that exposure to nevirapine was associated with protection from liver steatosis. Others have reported that nevirapine can effect favorable lipid changes [21-23], but efavirenz did not favorably affected lipid metabolism *in vivo*[23]. In a randomized clinical trial, lipid profiles improved when efavirenz was switched to nevirapine [23]. In our study, we confirmed a protective effect of nevirapine vis-à-vis lipid metabolism *in vivo,* including protection against liver steatosis.

Our study found that NRTIs such as AZT and d4T, and NNRTIs efavirenz were independent risk factors for liver steatosis in Chinese HIV/AIDS individuals, and the metabolic abnormalities may harbor a significant risk of developing metabolic syndrome, which indicated that regular monitoring of blood lipid levels and hepatic steatosis with Color Doppler Ultrasound was necessary after patients administered AZT or d4T and efavirenz. Therapeutic and prevention strategies may be of only limited clinical success, where avoiding the use of these medicines appeared to be most effective, and general recommendations include dietary changes, lifestyle modifications and switching antiretroviral therapy (replacing AZT or d4T with tenofovir, efavirenz with nevirapine).

Studies have reported that higher BMI is positively associated with liver steatosis, both in the general population and among HIV-infected patients. Higher BMI is an independent risk factor for NAFLD [24], but we did not see this association, which may be associated with genetic background in study population.

We found that Individuals with liver steatosis had lower level of triglyceride and fasting glucose, and lower prevalence of metabolic syndrome than those with non-steatosis (Table 2); and we also found that proportion of stavudine was significantly higher in non-steatosis group than that in liver-steatosis group (Table 4). Numerous studies have reported that stavudine had unfavorable effects of triglyceride and other metabolic disorders. Jericó et al. [25] reported that exposure of stavudine was associated with metabolic syndrome; Podzamczer [26] and Van et al. [27] also reported that concentration of triglyceride continued to increase after exposure of stavudine, which was consistent with our results.

Crum-Cianflone et al. [7] reported that liver enzyme abnormalities was not associated with NAFLD; however, Guaraldi et al. [16] reported that elevations in serum ALT level were an independent risk factor in a U.S. HIV-infected population. We did not find liver enzyme abnormalities to be associated with liver steatosis. Liver enzyme abnormalities were commonly seen in HIV-infected patients [28]. We found elevations in serum ALT level detected in patients from both liver steatosis and non-liver steatosis groups, indicated that liver enzyme abnormalities were associated with HIV infection and liver metabolic abnormalities *in vivo*. Our data suggest that elevations in serum liver enzyme levels were not specific for liver steatosis.

In this cohort, we found the prevalence of liver steatosis in HIV-infected patients with hypertriglyceridemia and past exposure to HAART was 46%, while only 5.5% of patients had hepatomegaly detected on ultrasound imaging. Lesi et al. [8] reported that, in African HIV-infected population with liver steatosis, 80% of patients have been detected hepatomegaly. Crum-Cianflone et al. [7] reported 63% of American HIV-infected population had hepatomegaly, suggesting that genetic factors and ethnic differences should be further investigated between Chinese and other races.

Our study limitations include: (1) the study design was a cross-sectional and causal relationships cannot be confirmed between risk factors and liver steatosis; (2) the determination of fatty liver disease was based on ultrasound imaging. Given that simple fatty liver and fatty hepatitis cannot be differentiated *in vivo*, we would have needed liver biopsies to definitively confirm liver steatosis [29].

We found that HIV-infected patients with hypertriglyceridemia and past HAART experience are prone to liver steatosis. In particular, HIV-infected patients who were using NRTIs should be screened and monitored over time as to their cholesterol panels and other signs of metabolic diseases.

## Abbreviations

3TC: Lamivudine; ALT: Alanine aminotransferase; AST: Aspartate aminotransferase; AZT: Zidovudine; BMI: Body metabolic index; BUN: Blood urea nitrogen; CK: Creati0ne kinase; Cr: Serum creatinine; d4T: Stavudine; EFV: Efavirenz; ELISA: Enzyme-linked immunosorbent assay; FPG: Fasting plasma glucose; HAART: Highly active antiretroviral therapy; HCV: Hepatitis C virus; HDL: High-density lipoprotein; HIV: Human immunodeficiency virus; LDL: Low-density lipoprotein; LPV/r: Lopinavir/Ritonavir; NAFLD: Nonalcoholic fatty liver disease; NVP: Nevirapine; TC: Total cholesterol; TCM: Traditional Chinese medicine; TDF: Tenofovir; TG: Triglyceride.

## Competing interests

The authors declare that they have no competing interests.

## Authors’ contributions

JX collected blood samples, participated laboratory tests and drafted the manuscript; NH collect blood samples; DY carried out statistical analysis; and HZ designed the project. All authors read and approved the final manuscript.
